# Screen Use Time and Its Association With Mental Health Issues in Young Adults in India: Protocol for a Cross-Sectional Study

**DOI:** 10.2196/39707

**Published:** 2024-07-16

**Authors:** Swasti Deshpande, Ashmeet Sachdev, Anwesha Maharana, Siddhesh Zadey, Surabhi P Dharmadhikari, Swati Ghate, Pawankumar Godatwar, Nisha Kumari Ojha, Sanjeev Sharma

**Affiliations:** 1 Association for Socially Applicable Research Pune India; 2 Department of Pediatrics Good Samaritan University Hospital West Islip, NY United States; 3 Centre for Mathematical Plasma-Astrophysics Katholieke Universiteit Leuven Leuven Belgium; 4 Dr. D. Y. Patil Dental College and Hospital Dr. D. Y. Patil Vidyapeeth Pune India; 5 GEMINI Research Center Duke University School of Medicine Durham, NC United States; 6 Department of Epidemiology Mailman School of Public Health, Columbia University New York, NY United States; 7 Babylon’s Newton Institute of Child and Adolescent Development Jaipur India; 8 Department of Roga & Vikriti Vijnana National Institute of Ayurveda Jaipur India; 9 Department of Kaumarbhritya-Balroga National Institute of Ayurveda Jaipur India; 10 National Institute of Ayurveda Jaipur India

**Keywords:** mental health, India, screen use, computer use, screen time, depression, stress, anxiety, low- and middle-income country, LMIC, questionnaire, survey, instrument, young adult, mental well-being, cross sectional, internet use, phone use, young adults

## Abstract

**Background:**

Screen use time has increased in the past decade owing to the increased availability and accessibility of digital devices and the internet. Several studies have shown an association between increased screen use time and mental health issues such as anxiety and depression. However, studies in the young adult population—a demographic with high screen use—and in low- and middle-income country settings are limited.

**Objective:**

This protocol describes a study that aims to measure self-reported screen use times and patterns in young adults (18-24 y) in India and assess if increased screen use time is associated with poorer mental well-being.

**Methods:**

This protocol describes a cross-sectional study of a pan-India, web-based convenience sample of young adults (18-24 y) with access to digital devices with a screen and a minimum of secondary school education. Participants will be recruited through people in the professional networks of the investigators, which includes pediatricians. The survey will also be distributed via the social media pages of our organization (X [X Corp], Instagram [Meta], Facebook [Meta], etc). Sociodemographic details will be collected through a questionnaire designed by the authors; screen use time and patterns will be assessed using an adaptation of the Screen Time Questionnaire to include data on different apps and websites used on digital devices; and mental health parameters will be gauged using the Warwick-Edinburgh Mental Well-Being Scale, Generalized Anxiety Disorder Scale, Perceived Stress Scale, and Patient Health Questionnaire. For statistical analysis, we will consider the following variables: (1) the primary independent variable is screen use time; (2) other independent variables include age, gender, residence: rural or urban, educational qualifications, employment status, stress associated with familial financial status, average sleep time, number of people living in a house or rooms in that house, BMI, substance use, and past psychiatric history; and (3) dependent variables include mental well-being, depression, anxiety, and perceived stress. To quantify the association between screen use time and mental health, we will perform a Bayesian multivariate multiple regression analysis that models the possibility of multiple alternative hypotheses while accounting for relevant sociodemographic covariables.

**Results:**

The survey instrument has been designed, and feedback has been obtained from the domain experts and members of our organization whose profile is similar to the potential study participants. The final data received after this study has been conducted will be analyzed and shared. As of January 2023, we have not yet initiated the data collection.

**Conclusions:**

Based on the findings of this study, we will be able to establish a correlation between device- and use-specific screen use time and various mental health parameters. This will provide a direction to develop screen use time and mental health guidelines among young adults.

**International Registered Report Identifier (IRRID):**

PRR1-10.2196/39707

## Introduction

Screen use time has steadily increased in the 21st century with increased availability of and easy accessibility to digital devices and the internet [1]. Adults and even children spend a large portion of their days on different screen-bearing devices including smartphones, tablets, laptops, computers, televisions, etc [1]. The recommended screen use time limit is considered to be 2 h/d [2]. However, studies have shown that screen use time has increased to as much as 7 h/d in groups aged 8-18 years in the United States [3]. As screens become increasingly integral to peoples’ social and professional lives, there is a rise in health-related concerns. Previous studies conducted in the United States and China have shown that increased screen use time has been associated with physical and mental health issues including increased incidence of anxiety and depression [2,4-6], sleep problems, and obesity [7]. Further, screen use times have only increased during the COVID-19 pandemic, making the issue a greater health research priority [8].

In 2021, the number of smartphone users in India reached 492.78 million, which accounts for about 16.93% of the total users worldwide [9]. Hence, it is timely to conduct studies to evaluate screen use time and its effects in low- and middle-income countries such as India with an increasing number of screens and large young (emerging) adult populations. Previously, screen use and its health impacts have been studied across multiple countries including the United States, China, and Canada. One study found the average daily screen use time to be between 1.73 hours on weekdays and 2.52 (SD 1.49) hours on weekends among children (mean age 7.4, SD 2.76 y) in Australia, which is higher than the recommended screen use time per Australian government guidelines. The guidelines suggest a screen use time of less than an hour for toddlers and preschoolers [10]. In Canada, the average screen use time was 1.78 (SD 1.52) h/d among adults. This study aimed to study the association between sedentary screen use time and neighborhood walkability, which showed that higher screen use time was found in individuals who did not have access to walkable neighborhoods [11]. The association between depression and screen use time is known to exist in different populations. For example, a study with college students in China has noted that the odds of depression were 1.76 times greater among those with over 2 hours of daily screen use time compared to those with lower use times [6]. Similarly, a study on Canadian youth found a significant correlation (*R*=0.212; *P*<.01) between sedentary total screen use time and depression severity [2]. Studies in India have focused on measuring screen use time and its correlation with various demographic factors, sleep problems, and mental health issues such as depression among adolescents [12,13]. Screen use time in children aged <5 years has been studied to assess its association with developmental delay [14]. These studies however do not use comprehensive activity-based screen use time. They also do not include the use of multiple mental health screening tools.

This study aims to measure the time spent on and patterns of screen use in individuals aged 18-24 years living in India. This study will probe into the different types of screen use–related factors such as types of devices, time and quality of engagement, and purpose of use. Young adults are of particular interest as behaviors adopted during developing years can persist and develop into chronic diseases [4,6]. This study includes a compilation of various existing mental health screening tools, which will help identify the presence of anxiety, depression, and stress. The hypothesis that increased screen use time is associated with various mental health problems including overall mental well-being, depression, anxiety, and perceived stress will be tested, while controlling for a wide range of possible confounders. A study such as this, with a comprehensive screen use time scale for different devices and compilation of multiple mental health screening tools, has not been conducted in India before. It could provide valuable data to help understand the public health impact and design preventive strategies for mental health issues due to increased screen use (Textbox 1).

**Design for this study’s plan.**

**Question**

- Is screen use time associated with mental health disorders (mental well-being, depression, anxiety, and perceived stress)?

**Hypothesis**

- On the contrary to a single alternative hypothesis in the frequentist approach, the Bayesian approach gives a probability distribution for multiple alternative hypotheses. Therefore, we aim to test the influence of all the independent variables on the dependent variables.

**Sampling plan (eg, power analysis)**

- We plan to perform a sequential Bayes factor (BF) design analysis. We will start with initial design priors for the variables, draw an initial sample size of N_eff_, and calculate the BF for the alternative models relative to the null model.

**Analysis plan**

- We will perform a Bayesian multivariate multiple regression analysis. We will use a default prior (normal distribution) from the RStudio package *rstanarm*, which is weakly informative, that is, the prior distribution is scaled based on the actual range of the outcome variables, and this will reduce the uncertainties in the posterior probabilities. Therefore, we plan to start with these default priors and then make some adjustments to the distributions as per our data set requirements.

**Interpretation given to different outcomes**

- Obtaining a BF_10_≥30 would imply that the screen use time, combined with other sets of predictors in that model, is strongly correlated with mental well-being, depression, anxiety, and perceived stress.

## Methods

### Ethical Considerations

This study will adhere to ethics regulations that are standard for noninterventional studies involving human participants in India. Ethics committee approval was obtained from the National Institute of Ayurveda, Jaipur, Rajasthan, India, on September 1, 2021 (IEC/ACA/2021/02-12), under NKO affiliated with the Department of Kaumarbhritya Balroga, National Institute of Ayurveda, Jaipur, Rajasthan, India. The approval has been signed by the Institutional Ethics Committee member secretary Dr Sumit Nathani (Associate Professor, National Institute of Ayurveda, Jaipur). Given that this study involves web-based data collection and storage, special attention will be given to data confidentiality and security. The confidentiality of each participant will be maintained throughout this study’s period as access to the raw data on participants will be available only to the members of this study’s team. Further, the questionnaire will not have any personal identifiers (name and date of birth) that would reveal the identity of the participants. The responses generated will be automatically transferred to a datasheet stored in the cloud-based service—Google Drive (a third-party trusted and commonly used data storage platform)—owned by the Association for Socially Applicable Research (ASAR) administration account and can be accessed only by this study’s team members.

### Pilot Data

No pilot data has been collected till now.

### Design

#### Recruitment and Data Collection

This will be a cross-sectional, web-based survey study conducted using a pan-India convenience sample. Participants will be recruited by investigators and people in their peer and professional networks. Additionally, the survey will be distributed through social media platforms that are popular in India such as WhatsApp (WhatsApp LLC), Facebook (Meta), Instagram (Meta), X (X Corp), etc. The survey will be posted on ASAR social media channels and those of collaborators regularly every 2 weeks for 6 months. The frequency will be adjusted based on the number of participants recruited in each such round. Concurrently, the social media channels will be modified and other web-based channels of dissemination will be explored. The survey will also be sent via email and SMS text messaging by investigators to people in their peer and professional networks. Our investigators are located in different regions in India, which will ensure nationwide coverage. A snowballing strategy will be used. Participants who have responded to the survey and people in the investigators’ networks will be requested to send the survey instrument further to people who might be eligible for study participation. Additionally, the survey will also be distributed through the higher education institutes that investigators routinely collaborate with.

The survey will be distributed in the form of Google Forms. Participants are expected to take about 10-15 minutes to complete the entire questionnaire. Once started, the participants will be able to resume the survey at any point later on, but we will recommend completing the survey in 1 week. Reminder emails will not be sent as the email IDs will be kept confidential. Additionally, participants will be directed to upload screenshots of their screen use time from their phones to assess objectivity. A step-by-step guide on how to upload the screenshot will be included in the questionnaire.

Data collection will be conducted for 12 months with the form being live for this duration. Eventually, survey data will be transferred to an external hard drive owned by the ASAR whose access is restricted to study investigators.

#### Inclusion and Exclusion Criteria

Participants for this study will be young adults aged 18-24 years from India who have access to digital devices with a screen (television, personal computer, laptop, tablet, or smartphone), those who have completed secondary school (10th standard passed), and those who have the basic knowledge of English reading and writing. Individuals aged younger than 18 years and aged older than 25 years will be excluded. Individuals who do not currently reside in India and have not resided in the last 12 months in the country, individuals who do not have access to digital devices with a screen, and those who have less than a secondary school education or have failed the 10th standard will be excluded.

#### Survey Instruments

All the survey questions will be made compulsory in the Google Form to avoid incomplete entries. The questionnaire consists of 114 questions across 4 parts. The first part is the introduction of this study and consent form for the participants. The second part is for the demographic and baseline details, including age, gender, residence (rural or urban), state, educational qualifications, employment status, the stress associated with familial financial status, average sleep time, number of people living in a house or rooms in that house, BMI, substance use, and past psychiatric history. This part of this study has been designed by the investigators, in line with other questionnaire-based surveys. The third part is the Screen Time Questionnaire [15]. The questionnaire from the cited study takes into consideration 2 types of screen use times. First, screen use time is the primary activity, which is defined as the main activity you are engaged in rather than using a television or other screens in the background while performing another activity such as cooking or exercising. Second, background screen use time is defined as the use of a television or another screen near you while performing other activities such as exercising, cooking, and interacting with family or friends. To obtain an accurate estimate of screen use time, we have modified the questionnaire by adding questions to measure differential screen use time. This will be measured by asking participants to enter the number of hours and minutes spent on screen-bearing devices (eg, televisions, mobile phones, laptops, etc) on various activities such as entertainment (eg, Netflix [Netflix], Amazon Prime [Amazon.com, Inc], YouTube [Google LLC], etc), gaming, social media (eg, WhatsApp, Facebook, X, etc), web-based classes or learning, or shopping websites (eg, Amazon, Myntra [Flipkart], etc). The fourth part is for mental health screening tools for well-being, depression, anxiety, and stress. Existing mental health screening tools will be used as they are without any modification [16]. The Warwick-Edinburgh Mental Well-Being Scale [17] will be used to assess mental well-being. This is a 14-item self-administered questionnaire, where each item is scored from 1 to 5 with a total score of 70. The interpretation of the scoring will be done according to the guidelines set by the National Health System Health Scotland [18]. The Patient Health Questionnaire will be used to assess the presence and severity of depression. This is a 9-item self-administered questionnaire, where each question is scored from 0 to 3. Patient Health Questionnaire scores of 5, 10, 15, and 20 represent the presence of mild, moderate, moderately severe, and severe depression, respectively [19]. The Generalized Anxiety Disorder Scale will be used to screen for the presence and severity of anxiety. This is a 7-item self-administered questionnaire, where each question is scored from 0 to 3. Scores of 5, 10, and 15 represent mild, moderate, and severe anxiety, respectively [20]. The Perceived Stress Scale [21] will be used to assess the perception of stress in an individual. It is a 10-item self-administered scale, where each item is scored from 0 to 4. Scores from 0 to 13 are considered low perceived stress. Scores from 14 to 26 are considered moderate stress. Scores from 27 to 40 are considered high perceived stress. At the end of the questionnaire, participants will be provided with mental health resources such as helplines, emergency numbers, etc.

The survey is coded using Google Forms. The questionnaires included in the survey have been reviewed by a psychiatrist and a psychologist. The first 3 parts of the survey have been adapted as per the feedback from ASAR members, who match the profile of the eligible participant population and guided the adaptation process. A copy of the survey instrument is presented in Multimedia Appendix 1.

### Statistical Analysis

#### Research Question and Hypothesis Testing

Our main research question is whether screen use time is associated with mental health issues such as mental well-being, depression, anxiety, and perceived stress. Before stating our formal hypotheses, we present the list of independent and dependent variables and their abbreviations that will be referred to in this section.

The primary independent variable or predictor is screen use time (T), which will be calculated per individual for the full week (5 weekdays, 5 weeknights, and 2 weekend days) as 5 × (T_per weekday_ + T_per weeknight_) + 2 × (T_per weekend day_) in minutes. Other independent variables include age (A), gender (G), residence (rural or urban; R), educational qualifications (E), employment status (EM), financial status (S), average sleep time (SL), number of people living in a house or rooms in that house (P), BMI, history of substance use (SU), and past psychiatric history (PPH). The outcomes or dependent variables will include mental well-being (MWB), depression (DEP), anxiety (ANX), and perceived stress (PS) as measured by the mental health screening tools mentioned above. Our null hypothesis is that there is no association between screen use time (T) and mental health issues (MWB, DEP, ANX, and PS), that is:

H0: [MWB, DEP, ANX, PS] ~ 1

Contrary to having a single alternative hypothesis in the frequentist approach, the Bayesian approach allows for probability distributions for multiple competing alternative hypotheses. Therefore, we aim to test the influence of all the abovementioned independent variables on the dependent variables through the following alternative hypotheses:

H1: [MWB, DEP, ANX, PS] ~ T

H2: [MWB, DEP, ANX, PS] ~ T + A

H3: [MWB, DEP, ANX, PS] ~ T + A + G

H4: [MWB, DEP, ANX, PS] ~ T + A + G + R

H5: [MWB, DEP, ANX, PS] ~ T + A + G + R + E

H6: [MWB, DEP, ANX, PS] ~ T + A + G + R + E + EM

H7: [MWB, DEP, ANX, PS] ~ T + A + G + R + E + EM + S

H8: [MWB, DEP, ANX, PS] ~ T + A + G + R + E + EM + S + SL + P

H9: [MWB, DEP, ANX, PS] ~ T + A + G + R + E + EM + S + SL + P + BMI

H10: [MWB, DEP, ANX, PS] ~ T + A + G + R + E + EM + S + SL + P + BMI + SU

H11: [MWB, DEP, ANX, PS] ~ T + A + G + R + E + EM + S + SL + P + BMI + SU + PPH

#### Sampling plan

We plan to perform a sequential Bayes factor (BF) design analysis. Since this study does not use the traditional frequentist null hypothesis testing, there is no need to determine the power or minimum sample size a priori. We will start with initial design priors for the variables, draw an initial sample size of N_eff_, and calculate the BF for the alternative models relative to the null model. We will then sequentially increase our sample size until the BF (relative to the null model, BF_10_) for the best-fit model, that is, the model with the highest posterior probability, becomes greater than or equal to 30. Obtaining a BF_10_≥30 would imply that screen use time is strongly correlated with mental health issues with a high posterior probability. Data collection will be continued until the end of 12 months. No optional stopping rule is required as it has been previously argued that under Bayesian formalism, the strength of the evidence is not associated with the decision to terminate data collection [22].

#### Analysis Plan

To ensure high data quality, we will use some basic exclusion checks before the analysis. The entire response entry (ie, participant) with at least 1 missing variable from any of the dependent and primary independent variables will be excluded. We will also exclude the responses that give entries with unrealistic screen use time (T), for example, exceeding 24 h/d. Data collection for different variables will be done in such a way that the candidates will have to fill in all the answers or choose out of multiple options provided. This would be useful for avoiding incomplete or missing data. The screenshots containing screen use time uploaded by the participants will be used as a secondary screen to further exclude the unrealistic responses.

Bayesian multivariate multiple regression analysis will be performed. The list of independent and dependent variables is provided under hypothesis testing. Before conducting the analysis, it has been assumed that the independent variables are not correlated with each other. After receiving the data from the survey, a correlation analysis between all the covariables will be performed first. The independent variables or covariables that have a strong correlation (Pearson *r*=0.7-1.0) with the primary independent variable will be dropped to reduce model complexity.

A default prior (normal distribution) from the RStudio (Posit) package *rstanarm* will be used, which is weakly informative [23], that is, the prior distribution is scaled based on the actual range of the dependent variables, and this will reduce the uncertainties in the posterior probabilities. Therefore, the analysis will be started with default priors with subsequent adjustments to the distributions as per the data requirements. The other backup prior that could be used is the standard Jeffreys-Zellner-Siow prior, which follows a Cauchy distribution and can model skewed real-world associations better.

After the prior distributions are finalized, there are 2 methods. First, these priors will be used to initialize the Bayesian multimodel inference using linear regression based on the Bayesian adaptive sampling methodology [24]. This methodology can be applied through a graphical interface tool, JASP (The JASP Team), which is based on the *BAS* package in R (R Foundation for Statistical Computing). This will enable prediction of the plausibility of each model, based on their posterior probability distributions. The BF for a particular model will be finally computed using this posterior probability relative to the posterior probability of the null model. Second, the *stan_glm* function in *rstanarm* can be continued to fit Bayesian generalized linear regression models. This package follows a Markov chain Monte Carlo technique to explore a desired posterior probability distribution. The advantage of using *rstanarm* is that it fits a wider range of models as compared to JASP.

## Results

The survey instrument has been created by designing a demographic questionnaire, compiling various existing mental health screening tools, and adapting an existing screen use time questionnaire. The instrument was reviewed by domain experts for feedback. This study will be funded by the National Institute of Ayurveda, Jaipur, India. As of January 2023, we have not yet initiated the data collection and the results obtained will be updated in this section accordingly.

## Discussion

### Principal Findings

This study aims to find the presence and magnitude of the association of screen use time used for different activities such as education, social media, web-based shopping, and entertainment with depression, anxiety, perceived stress, and general mental well-being while adjusting for confounders. We hypothesize that there is a positive correlation between screen use time and mental health issues such as depression, anxiety, and high levels of perceived stress. There is some support for this hypothesis from existing studies across different study populations and age groups.

### Comparison to Prior Work

A meta-analysis included 16 studies conducted in North America, Australia, and countries in Asia and Europe; the group aged 5-18 years shows a curvilinear dose-response between screen use time and the risk of depression, with or without increasing screen use time per day [3]. A study conducted in India among 16,292 adolescents and young adults in the group aged 10-19 years to examine the association between screen use time and sleep problems showed that screen use time increased with increasing age; with average screen use time being about 25 min/d for male and female individuals aged 12 years to about 150 min/d in male individuals and 50-100 min/d in female individuals aged 23 years [13]. A study involving 380 undergraduate college students in India showed that students with internet addiction (measured by Young’s Internet Addiction Test) had significantly higher depression, anxiety, and stress than those without internet addiction [25]. A community-based study with participants in the aged 10-19 years group in India found that the mean screen use time was 3.8 hours on weekdays and 3.9 hours on weekends [12]. This study also showed that there has been an increase in the use of digital devices apart from television such as mobile phones.

A meta-analysis including a total of 19 studies from the United States, Australia, Singapore, Sweden, China, Brazil, Ghana, India, South Korea, Russia, and Mexico showed an odds ratio of 1.28 (95% CI 1.17-1.39; *P*<.01) between screen use time–based sedentary behavior and depression [2]. A systematic review of reviews in the age group of <3 years, which included a total of 29 studies from the United States, countries in Europe, and Asia, showed that high screen use time is associated with low measured health-related quality of life and higher depressive symptoms [26]. A study establishing a relationship between screen use time, physical activity, and obesity in adolescents in the United States showed high predicted obesity incidence was associated with high screen use time [27]. A prospective study done in Denmark in 435 adolescents who were followed up for a period of 12 years showed that each additional hour per day spent watching television or screen viewing was associated with 1.64 (95% CI 1.18-2.27) and 1.58 (95% CI 1.18-2.12), respectively, greater odds of prevalent depression in young adulthood, and dose-response relationships were indicated [28]. A study among 926 US adults found that there were significant associations (*χ*^2^=14.96, *P*<.001) for heavy (median time: 17.5 h/d or 1050, IQR 900-1290 min/d) and moderate (median time: 11.25 h/d or 675, IQR 615-750 min/d) screen use with BMI compared to light (median time: 7 h/d or 420, IQR 328.50-480 min/d) use [7]. The relation between device-specific screen use time and health-related behavior has been studied by Vizcaino et al [7], which shows that heavy users (17.5 h/d) have the highest perceived stress scores.

### Strengths and Limitations

While studies showing a correlation between screen use time and mental health have been conducted, the strength of this study is that it studies device-specific screen use time and its relation with depression, anxiety, and perceived stress. The correlation between activity-specific screen use time and the mentioned mental health parameters will also be studied.

This study has the following limitations. First, this study uses a self-reported questionnaire due to which there might be some self-reporting bias. The questionnaire includes questions that ask the participants to recollect information based on their past experiences due to which there could also be recall bias. We are applying a secondary screen by asking participants to upload a screenshot of the screen use time settings on their phones. Since this question is not compulsory, all the participants might not upload it.

### Future Directions

The exponential rise in screen use time warrants further investigation of its effect on mental health. Studies done in India focus largely on adults and adolescents. The age group that will be studied in this protocol is unique. The findings of this protocol could be used to establish healthy activity-based screen use time. Strategies and guidelines for the prevention of mental health disorders can be established.

## Appendix

Multimedia Appendix 1 Example questionnaire.

## Abbreviations

ASAR: Association for Socially Applicable Research
BF: Bayes factor
