# Supplementary material for: Screen Use Time and Its Association With Mental Health Issues in Young Adults in India: Protocol for a Cross-Sectional Study
Source: JMIR Res Protoc. 2024 Jul 16;13:e39707. doi: 10.2196/39707 (PMC11289573; doi:10.2196/39707)
Supplement: Multimedia Appendix 1 [file resprot_v13i1e39707_app1.docx]

**Multimedia Appendix 1.** Example questionnaire.

**Screen exposure time and its association with mental health issues in young adults in India**

**PART 1:**

- Introduction to the study:

This study aims to measure the screen exposure times and patterns in young adults in India, study the hypothesis that increased screen exposure time is associated with poorer mental well-being and establish differential thresholds for screen exposure time. It is a cross-sectional study of a pan-India convenience sample of young adults (18-24 years) that have access to digital devices with a screen and a minimum of secondary school education (10th pass). Socio-demographic details will be collected through a questionnaire designed by the authors; screen exposure time and patterns will be assessed using an adaptation of the screen time questionnaire to include data on different applications and websites used on electronic devices; while mental health parameters will be gauged using the Warwick-Edinburgh mental well being scale, GAD-7 (a screening test for anxiety disorders), PSS-10 (measuring perceived stress levels) and PHQ-9 (to screen for depression). The details collected via this form will only be used for the purpose of this study. The identity of participants won't be revealed.

This study is being done by the Association for Socially Applicable Research (ASAR) in collaboration with the National Institute of Ayurveda (NIA). Ethics approval has been obtained from NIA.

Participation in this study is completely voluntary. For any queries please reach out to asarforindia@gmail.com.

**Part 2**

After reading the above details, do you consent to participate in the study?

Yes

No

**Part 3**

Demographic details

1. Age:

- 18
- 19
- 20
- 21
- 22
- 23
- 24
- Other: these would be discarded from data used for analysis

2. Sex

- Male
- Female
- Other: These would be discarded from data used for analysis

3. Place of residence:

- Rural
- Urban

4. State/Union Territories:

List Selection from all the states of India:

1. Andhra Pradesh
2. Arunachal Pradesh
3. Assam
4. Bihar
5. Chhattisgarh
6. Goa
7. Gujarat
8. Haryana
9. Himachal Pradesh
10. Jharkhand
11. Karnataka
12. Kerala
13. Madhya Pradesh
14. Maharashtra
15. Manipur
16. Meghalaya
17. Mizoram
18. Nagaland
19. Odisha
20. Punjab
21. Rajasthan
22. Sikkim
23. Tamil Nadu
24. Telangana
25. Tripura
26. Uttarakhand
27. Uttar Pradesh
28. West Bengal
29. Andaman and Nicobar Islands
30. Chandigarh
31. Dadra and Nagar Haveli and Daman & Diu
32. Delhi
33. Jammu & Kashmir
34. Ladakh
35. Lakshadweep
36. Puducherry

5. City of Residence: _________

6. Educational Qualifications:

- 10th Pass
- 12th Pass
- Graduate
- Post Graduate
- Diploma

7. Employment Status:

- Student
- Self-employed
- Employed in Government Sector
- Employed in Private Sector
- Homemaker
- Unemployed

8. What do you feel is the level of your/your family's financial stress today? [1]

(response options range from 1 [overwhelming stress] to 10 [no stress at all]

9. On average over the past 3 months, how many hours do you sleep at night? [2]

- Less Than 6
- 6-7
- 7-9
- 9-10
- Greater than 10

10. Do you have any past/present history of being on Psychiatric Medications/Treatment?

- Yes
- No
- Other _________

11. Number of people living in your house:

- 1
- 2
- 3
- 4
- 5
- 6
- 7
- 8
- 9
- 10
- Greater Than 10

12. No of total rooms in your house (e.g if 2 bedrooms, 1 hall and 1 kitchen write 4 rooms):

- 1
- 2
- 3
- 4
- 5
- 6
- 7
- 8
- 9
- 10
- Greater Than 10

13. Substance use question: In the last year have you ever drank (alcohol) or used drugs more than you meant to (alcohol, tobacco, marijuana, opioids, etc.)?

- Yes
- No

14. Have you felt you wanted or needed to cut down on your drug and drinking in the last year? [3]

- Yes
- No

15. Enter your height in metres (m)

     Enter your body weight in kilograms (kg)

**PART 4:**

Screen-time Questionnaire

For the following set of questions, ***primary activity*** is defined as the main activity you are engaged in rather than using a television/other screen in the background while performing another activity such as cooking or exercising.

| **Screen use on an average weekday**  Thinking of an average weekday (from when you wake up until you go to sleep), how much time do you spend using each of the following types of screen as the primary activity?       You must answer both hours and minutes. **If zero please type "0" in the box.** | | |
| --- | --- | --- |
|  | Hours | Minutes |
| Television |  |  |
| TV-connected devices (e.g. streaming devices, video game consoles) |  |  |
| Laptop/computer |  |  |
| Smartphone |  |  |
| Tablet |  |  |

| Out of the number of hours that you have entered for use on laptop/ smartphone/tablet; how much time do you spend for the activities mentioned below? Please enter the value in minutes and hours.  In case you’re uncertain about what time to enter follow the following instructions: 1. In your phone settings, go to Apps & Notifications ----> Screen Time. Then enter the values which are given for each of the apps mentioned below. | | | |
| --- | --- | --- | --- |
|  | | Hours | Minutes |
| Online learning/ online classes | |  |  |
| Work (Online meetings, presentations, etc) | |  |  |
| Gaming (Gaming consoles, Online games, etc) | |  |  |
| Entertainment | Netflix, Amazon Prime, Hotstar, etc. |  |  |
|  | Youtube |  |  |
|  | Sports streaming channels |  |  |
| Social Media | Facebook |  |  |
|  | Instagram |  |  |
|  | Snapchat |  |  |
|  | Twitter |  |  |
|  | Other social media |  |  |
| Messaging/ connecting with people | Whatsapp |  |  |
|  | Facebook Messenger |  |  |
|  | Telegram |  |  |
|  | Other messaging apps (Signal, text messages) |  |  |
| Social forums (eg, Reddit, Quora) | Reddit |  |  |
|  | Quora |  |  |
|  | Other social forums |  |  |
| Shopping websites (eg. Amazon, Myntra, Flipkart) | |  |  |
| News/ current events (eg. BBC news, TOI, Flipbpard) | |  |  |
| Fitness tracking apps (i.e. Any app that helps you track your calorie intake, fitness level, etc.)  (eg. Healthify me, etc.) | |  |  |
| Pornography | |  |  |
| Other applications: Camera, photo/video editing apps | |  |  |

| **Screen use on an average weekend day**  Now, thinking of an average weekend day (Saturday or Sunday), how many hours over the course of the whole day (from when you wake up until you go to sleep) do you spend using each of the following types of screen as the primary activity?  You must answer both hours and minutes. **If zero please type "0" in the box**. | | |
| --- | --- | --- |
|  | Hours | Minutes |
| Television |  |  |
| TV-connected devices (e.g. streaming devices, video game consoles) |  |  |
| Laptop/computer |  |  |
| Smartphone |  |  |
| Tablet |  |  |

| Out of the number of hours that you have entered for use on laptop/ smartphone/tablet for weekend use; how much time do you spend for the activities mentioned below? Please enter the value in minutes and hours.  In case you’re uncertain about what time to enter follow the following instructions: 1. In your phone settings, go to Apps & Notifications ----> Screen Time. Then enter the values which are given for each of the apps mentioned below. | | | |
| --- | --- | --- | --- |
|  | | Hours | Minutes |
| Online learning/ online classes | |  |  |
| Work (Online meetings, presentations, etc) | |  |  |
| Gaming (Gaming consoles, Online games, etc) | |  |  |
| Entertainment | Netflix, Amazon Prime, Hotstar, etc. |  |  |
|  | Youtube |  |  |
|  | Sports streaming channels |  |  |
| Social Media | Facebook |  |  |
|  | Instagram |  |  |
|  | Snapchat |  |  |
|  | Twitter |  |  |
|  | Other social media |  |  |
| Messaging/ connecting with people | Whatsapp |  |  |
|  | Facebook Messenger |  |  |
|  | Telegram |  |  |
|  | Other messaging apps (Signal, text messages) |  |  |
| Social forums (eg, Reddit, Quora) | Reddit |  |  |
|  | Quora |  |  |
|  | Other social forums |  |  |
| Shopping websites (eg. Amazon, Myntra, Flipkart) | |  |  |
| News/ current events (eg. BBC news, TOI, Flipbpard) | |  |  |
| Fitness tracking apps (i.e. Any app that helps you track your calorie intake, fitness level, etc.)  (eg. Healthify me, etc.) | |  |  |
| Pornography | |  |  |
| Other applications: Camera, photo/video editing apps | |  |  |

For the following set of questions, **background screen** is defined as the use of a television or another screen near you while performing other activities such as exercising, cooking, and interacting with family/friends.

Thinking about a regular weekday (Monday through Friday), on average, how many hours **over the course of the whole day** (from when you wake up until you go to sleep) are you exposed to background screen use? 

 *Example: If you exercise in the morning for one hour while watching the TV news, you use your smartphone for one hour while eating lunch and an additional 30 minutes while eating dinner, you would estimate that you are exposed to 2 hours and 30 minutes of background screen use per day.*

|  | Hours | Minutes |
| --- | --- | --- |
| Background screen use on a regular weekday |  |  |

Now we want to ask about background screen use **during the evening specifically**. On average, how many hours per evening (Monday through Friday) are you exposed to background screen use from when you return from work until you go to sleep?    
 *Example: If you regularly prepare dinner with the television on for one hour, and you keep the television on for an additional hour while using your smartphone for social media use, you can estimate that you are exposed to 2 hours of background screen use every evening.*

|  | Hours | Minutes |
| --- | --- | --- |
| Background screen use on a regular weeknight |  |  |

Now we want to ask about background screen use **during the weekend**. Thinking about a regular weekend day (Saturday or Sunday), on average, how many hours over the course of the whole day (from when you wake up until you go to sleep) are you exposed to background screen use?    
  
*Example: If you have the television on while you do some online shopping for two hours, and you keep the television on when friends come over to visit for an additional two hours, you can estimate that you are exposed to 4 hours of background screen use every evening.*

|  | Hours | Minutes |
| --- | --- | --- |
| Background screen use on a regular weekend day |  |  |

In order to get an accurate measurement of screen time, we are asking you to enter the total screen time as seen in your phone settings.

If you are an iOS user, in your phone, go to settings —-----> screen time. Please enter the total daily average screen time.

If you are an Android user , in your phone, go to settings —----> Digital Wellbeing & parental controls. Tap on the graph and enter the total screen time that you see for yesterday.

**PART 5:**

Warwick-Edinburgh Mental Well-being Scale:

Below are the statements regarding your feelings and thoughts. Please select the one that describes your experience of each one over the past 2 weeks.

| **Statement** | **None of the time** | **Rarely** | **Some of the time** | **Often** | **All the time** |
| --- | --- | --- | --- | --- | --- |
| I’ve been feeling optimistic about the future | 1 | 2 | 3 | 4 | 5 |
| I’ve been feeling useful | 1 | 2 | 3 | 4 | 5 |
| I’ve been feeling relaxed | 1 | 2 | 3 | 4 | 5 |
| I’ve been feeling interested in other people | 1 | 2 | 3 | 4 | 5 |
| I’ve had energy to spare | 1 | 2 | 3 | 4 | 5 |
| I’ve been dealing with problems well | 1 | 2 | 3 | 4 | 5 |
| I’ve been thinking clearly | 1 | 2 | 3 | 4 | 5 |
| I’ve been feeling good about myself | 1 | 2 | 3 | 4 | 5 |
| I’ve been feeling close to other people | 1 | 2 | 3 | 4 | 5 |
| I’ve been feeling confident | 1 | 2 | 3 | 4 | 5 |
| I’ve been able to make up my own mind about things | 1 | 2 | 3 | 4 | 55 |
| I’ve been feeling loved | 1 | 2 | 3 | 4 | 5 |
| I’ve been interested in new things | 1 | 2 | 3 | 4 | 5 |
| I’ve been feeling cheerful | 1 | 2 | 3 | 4 | 5 |

**PART 6: Generalized Anxiety Disorder- 7 (GAD-7) scale**

Over the last 2 weeks, how often have you been bothered by the following problems?

|  | Not at all | Several days | Over half the days | Nearly everyday |
| --- | --- | --- | --- | --- |
| 1.Feeling nervous, anxious, or on edge | 0 | 1 | 2 | 3 |
| 2.Not being able to stop or control worrying | 0 | 1 | 2 | 3 |
| 3. Worrying too much about different things | 0 | 1 | 2 | 3 |
| 4.  Trouble relaxing | 0 | 1 | 2 | 3 |
| 5. Being so restless that it’s hard to sit still | 0 | 1 | 2 | 3 |
| 6. Becoming easily annoyed or irritable | 0 | 1 | 2 | 3 |
| 7. Feeling afraid as if something awful might happen | 0 | 1 | 2 | 3 |

**Part 7: Perceived stress scale-l0**

The questions in this scale ask you about your feelings and thoughts during the last month. In each case, you will be asked to indicate by circling how often you felt or thought a certain way.

|  | Never | Almost Never | Sometimes | Fairly often | Very often |
| --- | --- | --- | --- | --- | --- |
| 1. In the last month, how often have you been upset because of something that happened unexpectedly? | 0 | 1 | 2 | 3 | 4 |
| 2. In the last month, how often have you felt that you were unable to control the important things in your life? | 0 | 1 | 2 | 3 | 4 |
| 3. In the last month, how often have you felt nervous and “stressed”? | 0 | 1 | 2 | 3 | 4 |
| 4. In the last month, how often have you felt confident about your ability to handle your personal problems? | 0 | 1 | 2 | 3 | 4 |
| 5. In the last month, how often have you felt that things were going your way? | 0 | 1 | 2 | 3 | 4 |
| 6. In the last month, how often have you found that you could not cope with all the things that you had to do? | 0 | 1 | 2 | 3 | 4 |
| 7. In the last month, how often have you been able to control irritations in your life? | 0 | 1 | 2 | 3 | 4 |
| 8. In the last month, how often have you felt that you were on top of things? | 0 | 1 | 2 | 3 | 4 |
| 9. In the last month, how often have you been angered because of things that were outside of your control? | 0 | 1 | 2 | 3 | 4 |
| 10. In the last month, how often have you felt difficulties were piling up so high that you could not overcome them? | 0 | 1 | 2 | 3 | 4 |

**PART 8: Patient health questionnaire-9 (PHQ-9)**

Over the last 2 weeks, how often have you been bothered by any of the following problems?

| Questions |  |  |  |  |
| --- | --- | --- | --- | --- |
| 1. Little interest or pleasure in doing things | 0 | 1 | 2 | 3 |
| 2. Feeling down, depressed, or hopeless | 0 | 1 | 2 | 3 |
| 3. Trouble falling or staying asleep, or sleeping too much | 0 | 1 | 2 | 3 |
| 4. Feeling tired or having little energy | 0 | 1 | 2 | 3 |
| 5. Poor appetite or overeating | 0 | 1 | 2 | 3 |
| 6. Feeling bad about yourself — or that you are a failure or have let yourself or your family down | 0 |  | 2 | 3 |
| 7. Trouble concentrating on things, such as reading the newspaper or watching television | 0 | 1 | 2 | 3 |
| 8. Moving or speaking so slowly that other people could have noticed? Or the opposite — being so fidgety or restless that you have been moving around a lot more than usual | 0 | 1 | 2 | 3 |
| 9. Thoughts that you would be better off dead or of hurting yourself in some way | 0 | 1 | 2 | 3 |

If any of the above problems were identified, how difficult have these made it for you to do your work, take care of things at home, or get along with other people?

Not Difficult At All

Somewhat Difficult

Very Difficult

Extremely Difficult

Should you score ≥ 5 on this questionnaire, we would recommend seeking help from a professional. Here are a few resources that might help you:

1. Kiran (1800–599–0019**)** is a national 24/7 toll-free helpline launched by the Ministry of Social Justice and Empowerment to help people with suicidal thoughts, depression, and other mental health issues.
2. AASRA (http://www.aasra.info/) (+91–22–27546669) is a 24-hours-a-day, 7 days a week nationwide voluntary, professional and confidential services.
3. National Emergency Number: 112

**Part 9:**

Thanks for filling the form.

We truly appreciate the time you took to fill the form out. We again would like to clarify that the information provided by you will be used solely for the study as mentioned in the beginning. Thank you very much once again! :)

If you are sure of the information you have filled in, click on the Submit button.

References:

1. Mugenda OM, Hira TK, Fanslow AM. Assessing the causal relationship among communication, money management practices, satisfaction with financial status, and satisfaction with quality of life. J Fam Econ Iss. 1990;11(4):343–60.
2. Chaput J-P, Dutil C, Sampasa-Kanyinga H. Sleeping hours: what is the ideal number and how does age impact this? Nat Sci Sleep. 2018 Nov 27;10:421–30.
3. McNeely J, Cleland CM, Strauss SM, Palamar JJ, Rotrosen J, Saitz R. Validation of Self-Administered Single-Item Screening Questions (SISQs) for Unhealthy Alcohol and Drug Use in Primary Care Patients. J Gen Intern Med. 2015 Dec;30(12):1757–64.
